# Pharmacokinetics studies of eugenol in Pacific white shrimp (*Litopenaeus vannamei*) after immersion bath

**DOI:** 10.1186/s12917-022-03145-3

**Published:** 2022-03-31

**Authors:** Yunyu Tang, Haixin Zhang, Guangxin Yang, Changling Fang, Cong Kong, Liangliang Tian, Xuanyun Huang

**Affiliations:** 1 East China Sea Fisheries Research Institute, Chinese Academy of Fishery Sciences, Jungong 300, Shanghai, 200090 P. R. China; 2 Jiang Xi Provincial Fisheries Research Institute, Fudayou 1099, Nanchang, 330039 P. R. China

**Keywords:** Eugenol, Pharmacokinetics, Anesthetic efficacy, Pacific white shrimp, Immersion bath

## Abstract

**Background:**

Eugenol is the most commonly used plant anesthetic to relieve the stressors during various aquaculture procedures. This study aims to investigate the pharmacokinetics of eugenol in Pacific white shrimp by immersion baths in a simulated transportation.

**Results:**

The pharmacokinetics of eugenol were firstly investigated in Pacific white shrimp by immersion baths of 300 mg L^− 1^ eugenol over 5 min (Treatment 1), 10 mg L^− 1^ eugenol during 24 h (Treatment 2) and a sequential immersion administration (Treatment 3). Concentrations of eugenol in hemolymph, hepatopancreas, and muscle were determined using Gas chromatography-tandem mass spectrometry (GC-MS/MS). After immersion bath of Treatment 1, the elimination half-life (t_1/2z_) values are 1.3 h and 11 h for hepatopancreas and muscles, indicating the rapid absorption and elimination of eugenol in shrimp. Under the Treatment 2 administration, the eugenol peak concentration is 6527.9 μg/kg in muscle, followed by 402.8 μg/kg in hepatopancreas, with the lowest concentration of 37.9 μg/L in hemolymph. Area under the curve (AUC_0-∞_) values lie in the order of muscle > hepatopancreas > hemolymph, suggesting that eugenol tends to accumulate in muscle by the immersion administration. Moreover, the average residence time (MRT_0-∞_) values of 38.6, 23.0 and 115.3 h for hemolymph, hepatopancreas and muscle are achieved, which may indicate that hepatopancreas is the main organ for elimination of eugenol. After combining the conditions in a sequential bath immersion of eugenol (Treatment 3), the maximum concentration (C_max_) values of eugenol are higher than those achieved in Treatment 2, indicating that accumulation of eugenol happened in haemolymph, hepatopancreas and muscle. In addition, the corresponding t_1/2z_ values are 4.7, 14.9 and 47.6 h, respectively, suggesting the faster elimination from the tissues following sequential administration. After the immersion bath, eugenol concentrations in muscle of Pacific white shrimp are lower than 2.5 mg/kg at 2 h, 48 h and 24.5 h in Treatment 1 ~ 3.

**Conclusions:**

A withdrawal period of 2 h, 48 h and 24.5 h following a 300 mg L^− 1^ of eugenol over a 5-min, 10 mg L^− 1^ eugenol concentration during a 24-h and combined conditions in a sequential immersion bath were suggested.

**Supplementary Information:**

The online version contains supplementary material available at 10.1186/s12917-022-03145-3.

## Background

Various fish and shellfishes are usually subjected to several stressors, including capture, handling, crowding, induced spawning and transportation in the aquaculture, which influence the physiological stress response and mechanical damage [1–3]. Hence, a variety of anesthetic agents are effective to reduce stress and risk of injuries during various aquaculture procedures [4–8]. Among them, eugenol is the most commonly used plant anesthetic, which has been demonstrated to be efficacy, safety and affordability as the crustacean anesthetic [9–11]. However, the efficacy of eugenol is closely related to various factors, resulting in the variation of the induction and recovery time [12–15]. Hence, the safety of eugenol should be evaluated when it is used in certain species [16–18].

The Pacific white shrimp (*Litopenaeus vannamei*) is one of the most popular shrimp species in China, with high quality animal protein for human consumption [19–21]. However, the rapid movement of shrimp tends to suffer damage and stress during capture, handling and crowding in transportation, resulting in high morbidity and low post-transport survival rate [8, 22, 23]. In this respect, there is no related research on the anesthetics efficacy and safety of eugenol in Pacific white shrimp. On the other hand, ready absorption, distribution to the tissues and rapid elimination are expected for an anesthetic, which is beneficial to the efficient recovery and avoid possible accumulation in the body [24, 25]. Until now, the study about the pharmacokinetics and residue elimination of eugenol in Pacific white shrimp has been not reported.

For now, the acceptable daily intake (ADI) of eugenol at 2.5 mg/kg was recommended by the Joint FAO/WHO Expert Committee on Food Additives (JECFA) [26]. Moreover, the maximum residue limit (MRL) of eugenol 0.05 mg/kg in Japan [27] and 0.1 mg/kg in New Zealand before 2005 [28]. Hence, considering the wide use of eugenol in shrimp, the pharmacokinetics, tissue distributions and elimination of eugenol are investigated in Pacific white shrimp for the first time, after immersion baths of 300 mg L^− 1^ eugenol over 5 min (Treatment 1), 10 mg L^− 1^ eugenol during 24 h (Treatment 2) and a sequential immersion (Treatment 3). These results firstly provide the reasonable rules of eugenol in Pacific white shrimp.

## Results

### Efficacy of eugenol

The induction and recovery data of high eugenol concentrations (100, 150, 200, 300 and 400 mg L^− 1^) in Pacific white shrimp are listed in Table 1. It is shown that induction and recovery time are related to the eugenol concentrations. In addition, five stages of induction and recovery from anesthesia are described in Table 2 [29]. The induction time of anesthesia stage 1, 2 and 3 decrease with the increasing concentrations of eugenol, while obviously increased time is observed for anesthesia recovery. Moreover, the lowest eugenol concentration of 100 mg·L^− 1^ caused deep anesthesia (stage 3) in 18.4 min, with the recovery time of 4.3 min. The shortest time for stage 3 (4.2 min) was observed at the maximum concentration of 400 mg·L^− 1^, while the recovery time exceeded 23 min. Recovery rates for the shrimps are 100% for 100, 150, 200 and 300 mg L^− 1^ eugenol after recovery for 7 days. However, 400 mg L^− 1^ eugenol resulted in 30% mortality of shrimps.

**Table 1:** Induction, recovery time and rates in Pacific white shrimp at different eugenol concentrations

Concentration	Induction Time/min			Recovery Time/min	Recovery rate/%
	1	2	3		
G1					
100	6.1 ± 1.7	11.4 ± 1.0	18.4 ± 1.9	4.3 ± 0.8	100
150	5.6 ± 1.6	7.9 ± 1.5	10.4 ± 1.8	6.1 ± 0.2	100
200	2.2 ± 0.6	4.2 ± 0.9	7.1 ± 1.7	9.1 ± 1.0	100
300	1.6 ± 0.2	2.7 ± 0.2	5.6 ± 1.1	9.5 ± 0.4	100
400	0.8 ± 0.2	1.9 ± 0.3	4.2 ± 0.7	23.6 ± 1.5	70
G2					
1	–	–	–	–	100
5	–	–	–	–	100
10	20.4 ± 1.8	–	–	1.2 ± 0.3	100
20	16.8 ± 1.5	29.7 ± 1.9	36.3 ± 1.2	2.1 ± 0.15^a^	100^a^
40	12.1 ± 1.4	22.5 ± 2.4	27.8 ± 1.7	2.9 ± 0.3^a^	100^a^

^a^The shrimps were moved to eugenol-free tank when they were observed in stage 3, and then the recovery and mortality were monitored

**Table 2:** Stages of anesthesia induction and recovery in shrimps

Stage	Behaviour description
Induction	
1	Partial or total loss of reaction in response to external stimuli except strong pressure, equilibrium is normal
2	Reaction to strong tactile and stimuli, partial loss of equilibrium
3	Failure to reflex activity and not reactive to strong external stimuli, complete loss of equilibrium
Recovery	
4	Start of erratic swimming, partial regained control of equilibrium
5	Complete regained control of equilibrium, attained an upright position and normal swimming behaviour

Relatively lower concentrations of eugenol (1, 5, 10, 20 and 40 mg L^− 1^) were investigated in Pacific white shrimp over a 24-h immersion bath (Table 1). There was no obvious anesthetic response observed in the eugenol concentration of 1 mg L^− 1^ and 5 mg L^− 1^, indicative of no sedative effects for shrimps in such low concentrations. The shrimps immersed in 10 mg L^− 1^ eugenol concentration induced sedative effects (stage 1) at 20.4 min and recovered in a rapid time about 1.2 min after a 24-h immersion bath. On the other hand, anesthetic doses of 20 and 40 mg L^− 1^ eugenol showed the effects on anesthesia (stage 3) during the immersion bath. Eugenol did not cause mortality when it evaluated at concentrations of 1, 5 and 10 mg L^− 1^ during 24-h exposure. In addition, the shrimps were not further immersed when the anesthesia phenomenon (stage 3) was observed during the immersion at eugenol solutions of 20 and 40 mg L^− 1^.

### LC-MS-MS analysis

In this study, the limit of detection (LOD) and quantification (LOQ) of eugenol by GC-MS/MS were 0.4 μg/kg and 1.0 μg/kg, respectively. The calibration plots were highly linear over a broad concentration ranges of 1.0 to 200 ng mL^− 1^ with the correlation coefficients (R^2^) of 0.9985 (Table 3). The recovery and precision were determined by spiking of eugenol at three different concentrations (one, five, and ten times of the LOD). The recoveries of eugenol were ranged from 86.6 to 117% at three spiking levels. The intra- and inter-day coefficients of variation were found below 10.3%. The corresponding data and GC-MS/MS chromatogram of eugenol are shown in Table S2 and Fig. S1.

**Table 3:** Linearity, calibration curve and correlation coefficient of eugenol

Linear ranges / ng mL^− 1^	Calibration Curve	Correlation Coefficient / R^2^
1.0 ~ 200	Y = 634.31*C-421.267	0.9985

### Pharmacokinetics

**Fig. 1:**
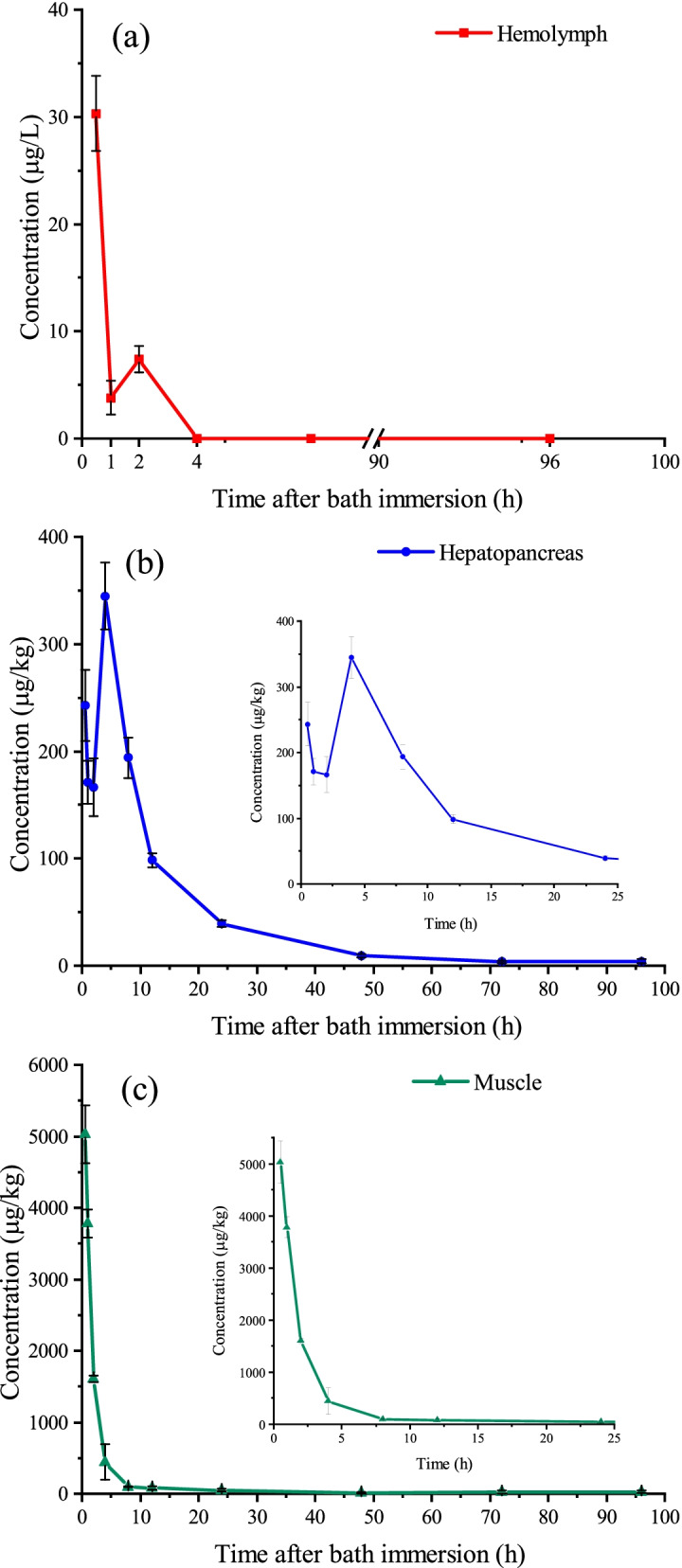
Concentration-time curves of eugenol in hemolymph (**a**), hepatopancreas (**b**) and muscle (**c**) after a 300 mg L^− 1^ immersion bath over 5-min period in Pacific white shrimp

Figure 1 shows the concentration-time curves of eugenol in hemolymph, hepatopancreas and muscle for Pacific white shrimp under Treatment 1. The eugenol concentrations showed a rapid decline from 30.3 μg/L to 3.8 μg/L in hemolymph during the first 1 h (Fig. 1). Furthermore, the eugenol cannot be detected at 4 h, indicative of a rapid elimination in hemolymph. For hepatopancreas, the maximum peak concentration was achieved at 4 h with value of 344.8 μg/kg, followed by a slow decline to the end of 96 h. In the muscle, the highest eugenol concentration was observed at the first sample point of 0.5 h, with the value of 5026.6 μg/kg. These observations indicate that the absorption of eugenol is fairly rapid in the shrimps. The highest eugenol concentrations rank in the order of muscle (5026.6 μg/kg) > hepatopancreas (344.8 μg/kg) > hemolymph (30.3 μg/L). It is noteworthy that the edible muscle tissues of eugenol concentrations are below the ADI value of 2.5 mg/kg at 2 h and the MRL of 0.05 mg/kg in Japan at 24 h. At the last sample point (96 h), the residual amounts of eugenol in hepatopancreas and muscle were 4.1 μg/kg and 26.0 μg/kg, respectively. The depuration of eugenol in Pacific white shrimp can be described by a first-order kinetic mode. The corresponding kinetic equations, goodness of fit (R^2^), slope (k) and half-lives (t_1/2z_) are listed in Table 4. The t_1/2z_ values were just 1.3 h and 11 h for hepatopancreas and muscle, respectively, indicating the fast eugenol elimination in shrimps after a high eugenol concentration immersion bath over a short time.

**Table 4:** kinetic equations, goodness of fit (R^2^), slope (k) and half-lives (t_1/2z_) of eugenol

Tissues	Equation	k	R^2^	t_1/2z_/h
Hepatopancreas	*C* = 0.2567e^-0.063t^	0.063	0.9608	1.3
Muscle	*C* = 5.4421e^-0.53t^	0.53	0.9737	11

**Fig. 2:**
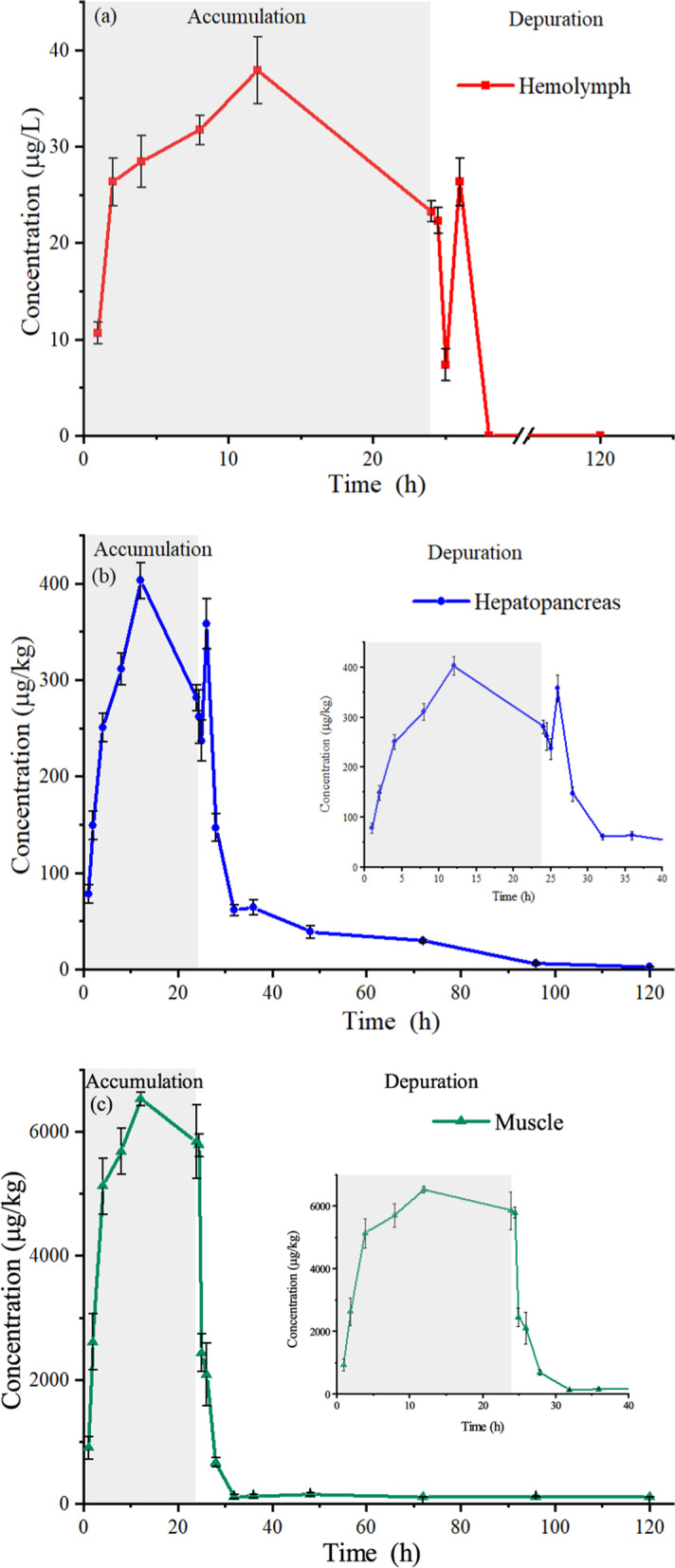
Concentration-time curves of eugenol in hemolymph (**a**), hepatopancreas (**b**) and muscle (**c**) following a 10 mg L^− 1^ immersion bath in Pacific white shrimp

The related concentration-time curves of eugenol under 10 mg L^− 1^ eugenol concentration during a 24-h immersion bath are shown in Fig. 2. The maximum concentrations (C_max_) in hemolymph (37.9 μg/L), hepatopancreas (402.8 μg/kg) and muscle (6527.9 μg/kg) were observed at 12 h, and then declined during the absorption period. The highest eugenol concentration was obtained in muscle, which was significantly higher than those in hepatopancreas and hemolymph. This observation is similar to that tested in Treatment 1. The second peak concentrations were observed at 26 h point with the values of 26.3 μg/L for hemolymph, as well as the values of 358.0 μg/kg at 26 h for hepatopancreas, which may be ascribed to the eugenol re-absorbed and re-distributed to tissues [30]. The eugenol concentration in muscle was achieved at 2.4 mg/kg at 25 h after drug exposure, which is lower than that of the ADI value. At the last sampling point (120 h), the eugenol concentrations were 2.5 μg/kg and 105.0 μg/kg in hepatopancreas and muscles, respectively. Eugenol was not detected at 28 h in hemolymph.

The estimated pharmacokinetics parameters are listed in Table 5. The elimination half-lives (t_1/2z_) of eugenol were 12.3, 21.4 and 79.3 h in hemolymph, hepatopancreas and muscle, respectively, indicating a long depletion time of muscle in shrimps. The AUC_0-∞_ values of eugenol in corresponding tissues were measured as 1518.7, 3921.5 and 41,942.9 μg h/L, respectively. This result suggests that eugenol tends to accumulate in the muscle by the immersion administration. In addition, the MRT_0-∞_ values of 38.6, 23.0 and 115.3 h for hemolymph, hepatopancreas and muscle were achieved, respectively, which indicated that hepatopancreas had an important effect on metabolism and elimination of eugenol.

**Table 5:** Pharmacokinetic parameters of eugenol in tissues following a 10 mg L^− 1^ immersion bath

Parameters	Unit	Hemolymph	Hepatopancreas	Muscle
C_max_	μg/kg	37.9	402.8	6527.9
T_max_	h	12	12	12
t_1/2z_	h	12.3	21.4	79.3
AUC_0-t_	ug/L*h	740.8	3814.7	22,174.1
AUC_0-∞_	ug/L*h	1518.7	3921.5	41,942.9
MRT_0-t_	h	12.6	20.0	30.5
MRT_0-∞_	h	38.6	23.0	115.3
Vz/F	L/kg	211.9	2365.929	818.449
CL/F	L/h/kg	6.6	76.5	7.2

Notes: C_max_ and T_max_ are the maximum concentration and the time of peak concentration; T_1/2_, elimination half-life; AUC, area under the concentration-time curve; MRT, average residence time; V_z_/F, apparent volume of the central compartment; CL/F, total clearance

**Fig. 3:**
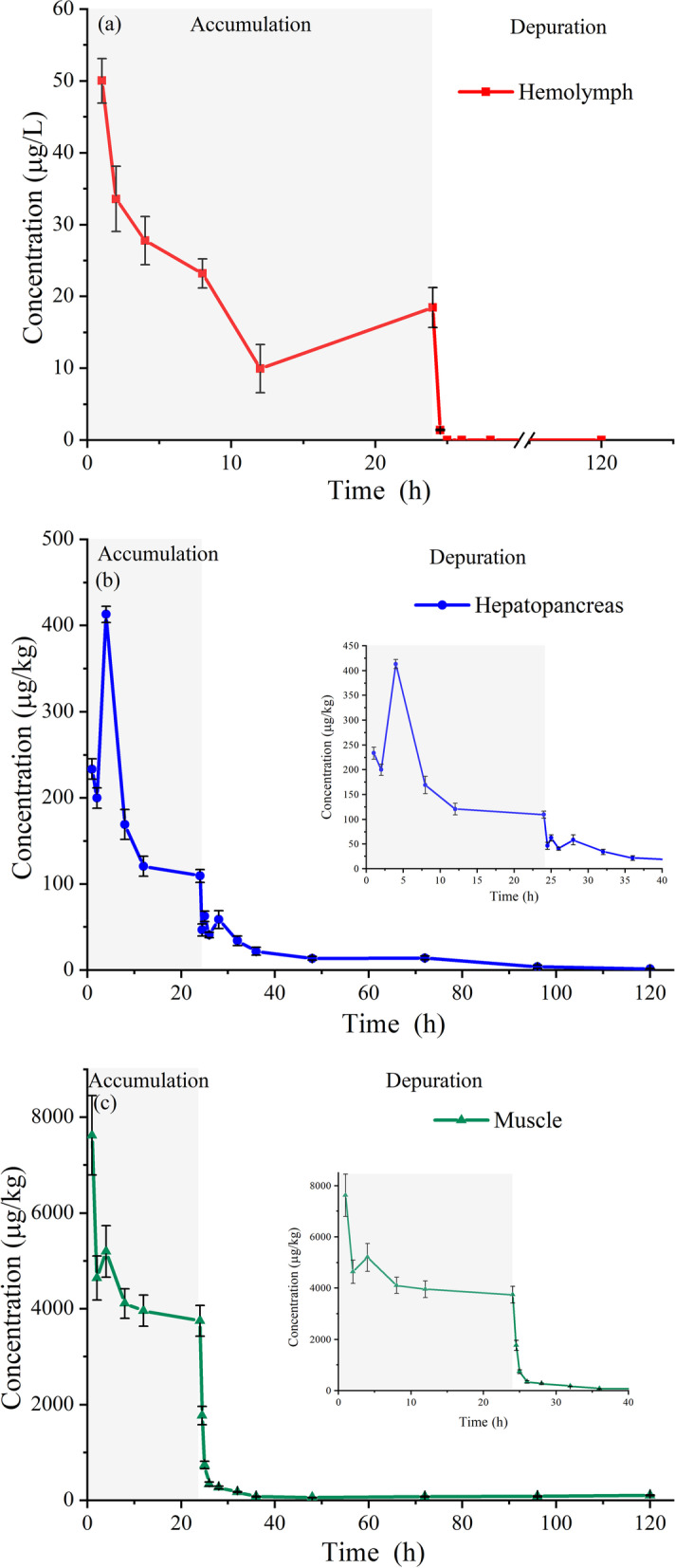
Concentration-time curves of eugenol in the hemolymph (**a**), hepatopancreas (**b**), and muscle (**c**) following a sequential immersion bath in Pacific white shrimp

The related concentration-time curves of eugenol under combined conditions in a sequential immersion bath are shown in Fig. 3. The eugenol concentrations in hemolymph, hepatopancreas and muscle exhibited a slowly decreasing trend in whole periods, with similar trend to those of Treatment 1. This observation may indicate that the high eugenol concentration dominates the trend of pharmacokinetics process in Pacific white shrimp in a sequential immersion bath. The C_max_ values of eugenol in hemolymph, hepatopancreas and muscle were 50.0 μg/L, 413.1 and 7623.0 μg/kg, respectively, peaked at 0.5 h except for hepatopancreas at 4 h. Moreover, the trends of depuration period for tissues are similar to those of Treatment 1 and Treatment 2, meaning that the depuration rule of the eugenol has minor relation with the eugenol concentration and the immersion time in Pacific white shrimp. The second peak concentrations were observed with the values of 18.4 μg/L at 24 h in hemolymph, 5200.0 μg/kg at 4 h in muscle, as well as the values of 62.5 μg/kg at 25 h and 58.7 μg/kg at 28 h in hepatopancreas, respectively. The eugenol concentration of 1.7 mg/kg in muscle was observed at 24.5 h. At the last sampling point (120 h), the eugenol concentrations were 1.5 μg/kg and 106.0 μg/kg in hepatopancreas and muscles, respectively. There were no values of eugenol detected at 1 h in hemolymph after the 24-h immersion. These observations indicate that the sequential bath immersion is beneficial to decrease the residual eugenol concentrations in shrimps and increase the metabolism.

The calculated PK parameters are presented in Table 6. The t_1/2z_ values of eugenol were 4.7 h, 14.9 h and 47.6 h in hemolymph, hepatopancreas and muscle, respectively, which were more rapid than those in Treatment 2, indicative of faster eugenol elimination from the tissues following a sequential immersion administration. The corresponding AUC_0–∞_ values were 481.8, 1342.2 and 22,179.5 μg/L*h, respectively, revealing that the accumulation of eugenol in the tissues increased at the sequential immersion bath administration. The MRT_0-∞_ values were 10.9 h, 28.4 h and 104.1 h in corresponding tissues.

**Table 6:** Pharmacokinetic parameters of eugenol in tissues following a sequential immersion bath

Parameters	Unit	Hemolymph	Hepatopancreas	Muscle
C_max_	μg/kg	50.0	413.1	7623.0
T_max_	h	1	4	1
t_1/2z_	h	4.7	14.9	47.6
AUC_0-t_	ug/L*h	471.8	1311.7	11,008.7
AUC_0-∞_	ug/L*h	481.8	1342.2	22,179.5
MRT_0-t_	h	10.5	26.4	42.6
MRT_0-∞_	h	10.9	28.4	104.1
Vz/F	L/kg	141.8	4803.2	928.9
CL/F	L/h/kg	20.8	223.5	13.5

Notes: The meanings of C_max_, T_max_, AUC, T_1/2z_, MRT, Vz/F and CL/F are the same as those in Table 5

## Discussion

### Efficacy of eugenol

Anesthetics have been widely used for aquaculture animals to avoid injuries and stress, ensuring the good quality and safety during the handling process and transportation [31–33]. Thus, it is important to assess the efficacy of the anesthetic dose in different aquaculture species [12, 34]. In recent years, eugenol has been regarded as an effective anesthetic in many crustaceans [18, 35]. However, there are no recommended eugenol does for crustaceans due to the lack of such researches. Hence, the first objective of present study is designed to evaluate the suitable immersion time and dosage for anesthesia and sedation in Pacific white shrimp (*L. vannamei*).

Generally, anesthesia at high eugenol concentrations with short bath time is required under the manipulation procedures. However, high concentrations may result in the mortalities to some extent [5]. Thus, the safety of the high eugenol concentrations in Pacific white shrimp was assessed, which revealed the high survival rates and smooth induction along the stages, with deep anesthesia. Accordingly, an ideal anesthetic should induce anesthesia with 3 min and promote an anesthetic recovery time within 5 min [36–38]. As a result, 300 mg L^− 1^ of eugenol over a 5-min immersion bath proved to have good anesthetic effects in Pacific white shrimp without mortality. The recorded time for induction and recovery were 5.6 min and 9.5 min, respectively. Li [8] found that eugenol concentrations above 300 μL L^− 1^ may cause higher mortality on grass shrimp (*Palaemonetes sinensis*) with the temperature above 20 °C. The post-larvae was rapidly anesthetized in 4.1 min at 175 μL L^− 1^ eugenol concentration in Pacific white shrimp [39]. In our study, the anesthesia time decreases with the increase of eugenol concentrations, while the recovery time showed the opposite tendency. Similar observations were obtained in *Lateolabrax maculatus* [40], *Garra rufa* [15], *Cyprinus carpio* [4] and *Barbus grypus* [16].

On the other hand, anesthetics at low concentrations are used for transportation as sedative effects, which is effective to reduce the stress and agitation [41, 42]. Considering that eugenol used for long-term sedation should avoid deep anesthesia, 1 ~ 40 mg L^− 1^ eugenol concentrations were investigated in Pacific white shrimp. As a result, 10 mg L^− 1^ eugenol over a 24-h immersion bath was suitable for sedation in the simulative transportation, with a 1.2 min recovery time. In this respect, it was reported that 20 μL L^− 1^ eugenol was sufficient to cause sedation for the post-larvae of *L. vannamei* over 6 h and recovered in 5 ~ 10 min [39]. Compared to the post-larvae of *L. vannamei*, the post-larvae of *Fenneropenaeus indicus* showed high mortality rate under 4 μL L^− 1^ eugenol for 12 h transportation, implying that it was more sensitive to eugenol [43]. In addition, the eugenol concentration of 53.0 mg L^− 1^ was used for hybrid catfish to achieve a higher safety margin and reduce mortality [44]. He [40] reported that obviously low survival rates were observed for spotted sea bass at 10 and 15 mg L^− 1^ of eugenol concentrations after 24-h transportation. Therefore, the range of eugenol should be optimized for the transportation of Pacific white shrimp, which is obviously different to fish and other shrimp species. As a result, 300 and 10 mg L^− 1^ are effective to induce deep anesthesia and sedation for shrimps during the simulated handling and transportation.

### Accumulation and clearance of eugenol

As anesthetics are widely used on aquatic animals, the pharmacokinetic properties and residue elimination of anesthetics attract the attention of the world [36, 45]. Eugenol has been found to efficiently induce anesthesia in crustaceans [22]. However, studies on the residue elimination of eugenol are limited in rainbow trout [46], Japanese Flounde [47] and grass carp [48]. Till now, there is no related research on pharmacokinetic of eugenol in shrimps. Lots of pharmacokinetics studies in shrimp were related to the antibacterial agents, such as oxytetracycline in white shrimp and black tiger shrimp [49, 50], sulfamethoxazole and enrofloxacin in Pacific white shrimp [19, 51], sulphamethazine in *Fenneropenaeus chinensis* shrimp [52] and oxolinic acid in kuruma shrimp [53]. Hence, in the present study, pharmacokinetics and residue eliminations of eugenol in Pacific white shrimp were firstly investigated using GC-MS/MS. Meanwhile, the accumulation and depuration of eugenol in hemolymph, hepatopancreas and muscle of shrimps were investigated after exposure to three anesthetic treatments (Figs.1, 2 and 3). In addition, the pharmacokinetic data were analysed by a non-compartmental model due to its concision and comparability.

Three hundred microgram L^− 1^ of eugenol over a 5-min immersion bath (Treatment 1) proved to have good anesthetic effects in the manipulation procedures of shrimps. The eugenol was depurated in hemolymph after 2 h and the value decreased from 5026.6 μg/kg to 81.2 μg/kg in muscle at about 12 h (Fig. 1). In this respect, Zhao [48] reported that the eugenol concentrations decreased rapidly from 24.63 mg/kg to 2 mg/kg at about 12 h in grass carp. Similar results were observed in silver perch, which exhibited an obviously decrease from 16 mg/kg to 3 mg/kg within 6 h [54]. The accumulation period was not monitored, which may be attributed to no sampling time points taken in the first 30 min after the immersion bath. These observations suggest that eugenol metabolism is rapid in a short time.

During a long-term immersion, 10 mg L^− 1^ eugenol produced sedation effects in Pacific white shrimp, which was sufficient to transport the shrimps (Treatment 2). As shown in Fig. 2 and Table 5, the C_max_ values lie in the order of hemolymph < hepatopancreas < muscles, which is consistent with that in Treatment 1. Vorbach [55] showed that the concentrations of oxytetracycline in fish muscle and blood were 27.17 mg/L and 16.54 mg/L after 12 h immersion at the dose of 25 mg/L, respectively. The C_max_ of enrofloxacin in rainbow trout were found to be 0.51 μg/g and 0.40 μg/mL in muscle and plasma after bath immersion [56], respectively. These observations indicate higher drug absorption in the muscle during the immersion bath administration. Moreover, it could be found that the C_max_ values are relatively higher than those obtained at 0.5 h in Treatment 1. This observation may be ascribed that the Pacific white shrimps kept at stage 1 in Treatment 2 and totally recovered to normal at 0.5 h in Treatment 1 (Table 1). In addition, elimination half-life (t_1/2z_) is often discussed as an important pharmacokinetic parameter. The t_1/2z_ values of oxytetracycline were calculated to be 14.89 h and 23.53 h in hepatopancreas and muscles [57]. Fu [58] showed t_1/2z_ of oxolinic acid were 5.82 h and 9.99 h in above tissues at the dose of 10 mg/kg in shrimps, respectively. In the present study, the t_1/2z_ values of eugenol are fitted to be 21.4 h and 79.3 h in the hepatopancreas and muscle after a long-term immersion (Table 5), which is good consistent with the results described above, suggesting a faster depuration in hepatopancreas than that of muscle. Moreover, the t_1/2z_ of eugenol in shrimp is obviously lower than those of other chemical compounds, such as sulfamethoxazole in Pacific white shrimp (13.76 h) [51], enrofloxacin in Pacific white shrimp (19.8 h) [21], oxytetracycline in female and male *F. chinensis* (19.58 and 16.11 h) [59] and oxolinic acid in black tiger shrimp (17.7 h) [60]. These results suggest that eugenol has a more rapid elimination in shrimp.

With the purpose to simulate the process of handling and transportation, the short-term and long-term treatments were combined in a sequential immersion bath (Treatment 3) for Pacific white shrimp. The corresponding concentration-time curves and pharmacokinetic parameters are illustrated as Fig. 3 and Table 6. The C_max_ values of eugenol are 50.0 μg/L, 413.1 μg/kg and 7623.0 μg/kg in haemolymph, hepatopancreas and muscle, which are higher than those achieved in Treatment 2, indicating that eugenol accumulates in the haemolymph, hepatopancreas and muscle. Compared to the peak concentrations in single-dose administration, higher values could be observed in multiple-dose administration in a lot of drugs. The oxytetracycline peak concentrations were 14.02, 8.51, 4.17, 3.84 μg/g in plasma, muscle, liver and kidney in *Pelteobagrus fulvidraco* after the multiple-dose oral administration, which were much higher than 1.46, 1.39, 3.48 and 2.90 μg/g after the single dose oral administration [61]. Two point seventy-seven microgram per gram of enrofloxacin was detected in muscle of giant freshwater prawns at the dose of 5 g/kg for 5 days, while peak muscle concentration was 1.98 μg /g at a single dosage of 10 mg/kg [62]. These observations indicate that most drugs tend to accumulate in hemolymph, hepatopancreas and muscle. In spite of the higher eugenol peak concentration in Treatment 3, the residual amounts of eugenol in hemolymph, hepatopancreas and muscle are obviously lower than that in Treatment 2 after 24 h. Additionally, it is noteworthy that the t_1/2z_ value in the shrimp is lower than that of 19.79 h in grass carp [48], 12.14 h in rainbow trout [54] and 8.08 h in Japanese Flounder [47]. These observations suggest that the elimination rate of eugenol in shrimp is more rapid than that in fish. Similar results were found in sulfamethoxazole, oxytetracycline and trimethoprim eliminations in shrimp respect to those in fish [51], which may be ascribed to the especial anatomy and physiology [57] owning to the open circulatory system and dispersion type liver in shrimp, resulting in the rapid drug excretion through the haemolymph and hepatopancreas. In addition, the elimination half-life values in Treatment 3 are lower than those in Treatment 2, which may be ascribed to the faster elimination rate with a higher peak concentration, due to the elimination of eugenol fitting the first-order dynamic equation. Besides, the CL/F values of eugenol are 20.8, 223.5 and 13.5 L/h/kg in hemolymph, hepatopancreas and muscle, indicative of a faster elimination rate in hepatopancreas. In this aspect, a lot of drugs were reported to eliminate fast in the hepatopancreas of crustaceans, such as enrofloxacin in *Scylla serrata* [63] and oxolinic acid and oxytetracycline in Pacific white shrimp [20, 58], which may be ascribed that the hepatopancreas is the main organ in metabolism and elimination for crustaceans, different from the liver or kidney for vertebrates [64, 65]. On the other hand, it is noteworthy that the residual eugenol concentrations of 1.60, 2.43 and 1.77 mg/kg are observed in muscles after 2 h during the short-term immersion (Treatment 1), 48 h in the long-term immersion (Treatment 2) and 24.5 h in the sequential immersion administrations (Treatment 3), which are both lower than that of 2.5 mg/kg recommended by the JECFA for acceptable daily intake. Thus, 2 days is recommended as a minimum withdrawal period of Pacific white shrimp after a long time eugenol exposure for human consumption.

## Conclusion

In summary, eugenol concentrations of 10 and 300 mg L^− 1^ are efficacious and suitable for sedation and anesthesia in Pacific white shrimp (12 ± 2.0 g), which is beneficial to mitigate the stress response after handling and transportation, improving biochemical responses and reducing mortality. Moreover, pharmacokinetics and depuration of eugenol in Pacific white shrimp (12 ± 2.0 g) by the immersion bath were firstly investigated in the present study. The pharmacokinetics of eugenol indicated a rapid absorption and elimination in shrimp. With the aim to ensure the safe consumption of Pacific white shrimp, a withdrawal period of 2 h, 48 h and 24.5 h following a 300 mg L^− 1^ of eugenol over a 5-min, 10 mg L^− 1^ eugenol concentration during a 24-h and combined conditions in sequential immersion bath were suggested. Moreover, the potential affects/concentrations to different size of shrimps will be evaluated in our future work.

## Methods

### Chemicals and reagents

Eugenol analytical standards (purity, 99.6%) was purchased from Sigma-Aldrich Corporation (St. Louis, MO, USA). Eugenol (purity, 98.5%) was purchased from Sinopharm Chemical Reagent Co., Ltd. (Shanghai, China). Methanol, acetonitrile and acetic acid were liquid chromatography grade (Merck, Darmstadt, Germany). De-ionized water was produced from a Milli-Q water purification system (Millipore Ltd., Bedford, MA, USA). The Bond Elut-PH Cartridges (500 mg/3 mL) was obtained from Agilent Technologies (Santa Clara, CA, USA).

### Experimental animals

Approximately 1500 individuals (12 ± 2.0 g) of Pacific white shrimp were purchased from Shanghai nongcang aquaculture Co., Ltd. (Shanghai, China) in July 2020. They were divided into 6 tanks (800 L capacity) with a circular flow system with fully aerated tap water. Aeration was provided by a Roots-type blower and air stones. The shrimps were held for 21 days at 20 °C ± 0.5 °C before the experimentation and fed 2% of body weight every 12 h. Six shrimps were chosen randomly to confirm the absence of eugenol in hemolymph, hepatopancreas and muscle.

### Anesthetic efficacy of eugenol

Anesthetic efficacy of high eugenol concentrations by an immersion bath was investigated. Eugenol dissolved in 95% ethanol (ratio of eugenol to ethanol, 1:9). 100, 150, 200, 300 and 400 mg L^− 1^ eugenol were evaluated on Pacific white shrimp at 20 ± 0.5 °C, respectively. Every 10 shrimps were exposed to each concentration, monitored the behaviour responses and the anesthesia time. After shrimps exhibited the behaviour of stage 3 (Table 2), they were moved to recovery tanks to monitor recovery and mortality. Shrimps were regarded as eugenol free at stage 5. The survival rates were observed every 24 h in the following 7 days.

Anesthetic efficacy of low eugenol concentrations by a 24-h immersion bath was evaluated. Ten shrimps were individually maintained in five eugenol concentrations at 1, 5, 10, 20, 40 mg L^− 1^ for 24 h immersion bath at 20 ± 0.5 °C, respectively, and recorded the phenomenon for 1 min.

### Pharmacokinetic study

Pacific white shrimps were immersed in a solution of 300 mg L^− 1^ eugenol for a period of 5 min at 20 ± 0.5 °C, then rinsed thoroughly with purified water and transferred to anesthetic-free tank (I.D. 120 cm × 70 cm). The sampling time was set at 0.5 h, 1 h, 2 h, 4 h, 8 h, 24 h, 48 h, 72 h and 96 h following the eugenol immersion bath (Treatment 1).

The shrimps were held in a eugenol solution of 10 mg L^− 1^ for 24 h at 20 ± 0.5 °C, and sampled at several time points of 1 h, 2 h, 4 h, 8 h, 12 h and 24 h during the immersion bath. After 24 h, the shrimps were rinsed thoroughly with purified water and transferred to anesthetic-free tank (I.D. 120 cm × 70 cm). Then, samples were further taken at 24.5 h, 25 h, 26 h, 28 h, 32 h, 48 h, 72 h, 96 h and 120 h in the following eugenol free immersion bath (Treatment 2).

When the Pacific white shrimps showed a complete recovery (stage 5) from Treatment 1, the shrimps were transferred to the condition of Treatment 2. The collected time points were the same as the Treatment 2 (Treatment 3).

### Sample collection

At each sampling time point, 30 shrimps were randomly chosen to get hemolymph, hepatopancreas and muscle. Hemolymph were taken from the pericardial cavity with a 1 mL heparinised syringes. Then, hepatopancreas and muscles were collected and stored at − 80 °C. At each time point, five shrimps were considered as one sample.

### Sample preparation

Hemolymph (0.5 mL) was added to 0.5 mL of ethyl acetate in a 2.0 mL centrifuge tube. The sample was vortex-mixed for 2 min, followed by centrifugation at 10,000 r/min for 10 min at 4 °C. Then, the supernatant was collected and filtered through 0.22 μm filters.

Hepatopancreas and muscle (2.00 ± 0.05 g) were homogenized in 5 mL of n-hexane, followed by vortexing for 1 min. After that, the mixture was centrifuged at 4500 r/min for 10 min and the supernatant was moved to another centrifuge tube. The remaining tissues were extracted again and the supernatants were combined. The Bond Elut-PH extraction cartridges were pre-conditioned with 3 mL of ethyl acetate and 3 mL of n-hexane. The supernatants passed through the cartridge, and then washed with 5 mL of n-hexane. At last, the retained eugenol was eluted by 2 mL of ethyl acetate. After filtration with 0.22 μm filters, the samples were ready for gas chromatography-mass spectrometry (GC-MS/MS) analysis.

### Analytical procedures

The GC-MS/MS includes a Themo Scientific GC system (US) and a Thermo TSQ Quantum Ultra triple-quadrupole mass spectrometer with electron ionization (EI) mode. The chromatographic separation was performed with a DB-17MS capillary column (30 m × 0.25 mm × 0.25 μm) made by Agilent Technologies. Helium (99.999% pure; Air Liquide) acted as carrier gas with the flow rate of 1.0 mL min^− 1^. The injector and the GC-MS interface temperatures were set to 260 °C and 280 °C, respectively. The initial column temperature was 80 °C and maintained for 2 min. Then it was increased to 250 °C at a rate of 25 °C min^− 1^ and held for 5 min. Finally, the temperature ramped to 280 °C at a rate of 25 °C min^− 1^ and held for 17 min. The injection volume of the samples was 1 μL and the solvent delay time was 180 s.

The ion source and quadrupole temperatures were 230 °C and 150 °C, respectively. The electron ionization energy of the mass selective detector was 70 eV. The major fragment ions used to quantify and qualify eugenol are shown in Table S1.

### Statistical and pharmacokinetic calculations

Data were analyzed as mean ± standard deviation (SD). Pharmacokinetic analysis was analyzed using DAS version 3.0 (Mathematical Pharmacology Professional Committee of China). The pharmacokinetic parameters were calculated based on the statistical moment theory for eugenol. The following PK parameters were calculated: the maximum concentration (C_max_), time to reach C_max_ (T_max_), area under the concentration-time curve (AUC), average residence time (MRT), elimination half-life (t_1/2z_), extensive apparent volume of the central compartment (V_z_/F) and total clearance (CL/F).

## Supplementary Information

**Additional file 1: Table S1.** Detection parameters of eugenol for mass spectrometer. **Table S2.** Recovery, accuracy and precision of eugenol in feed samples (*n* = 6). **Fig. S1.** Chromatograms of eugenol in spiked fish feed sample (2 μg/kg).

## Data Availability

The datasets used and/or analyzed during the current study are available from the corresponding author on reasonable request.

## Abbreviations

ADI: Acceptable daily intake
JECFA: The Joint FAO/WHO Expert Committee on Food Additives
MRL: The maximum residue limit
LOD: The limit of detection
LOQ: The limit of quantification
C_max_: The maximum concentration
T_max_: Time to reach C_max_
AUC: Area under the concentration-time curve
MRT: Average residence time
t_1/2z_: Elimination half-life
V_z_/F: Extensive apparent volume of the central compartment
CL/F: Total clearance
