# Supplementary material for: Pharmacokinetics studies of eugenol in Pacific white shrimp (Litopenaeus vannamei) after immersion bath
Source: BMC Vet Res. 2022 Mar 31;18:122. doi: 10.1186/s12917-022-03145-3 (PMC8969250; doi:10.1186/s12917-022-03145-3)
Supplement: Supplementary file 1 — Additional file 1: Table S1. Detection parameters of eugenol for mass spectrometer. Table S2. Recovery, accuracy and precision of eugenol in feed samples (n = 6). Fig. S1. Chromatograms of eugenol in spiked fish feed sample (2 μg/kg). [file 12917_2022_3145_MOESM1_ESM.docx]

**Pharmacokinetics studies of eugenol in Pacific white shrimp (*****Litopenaeus vannamei*) after immersion bath**

Yunyu Tang^a,†^, Haixin Zhang^b,†^, Guangxin Yang^a^, Changling Fang^a^, Cong Kong^a^, Liangliang Tian^a^, Xuanyun Huang^a,^*

*^a^* East China Sea Fisheries Research Institute, Chinese Academy of Fishery Sciences, Jungong 300, Shanghai 200090, P. R. China.

^b^ Jiang Xi Provincial Fisheries research institute, Fudayou 1099, Nanchang 330039, P. R. China.

* Corresponding author: Xuanyun Huang, E-mail: hxyseven@163.com

^†^ These authors contribute equally to this work.

Table S1 Detection parameters of eugenol for mass spectrometer

| Retention time/min | Quantification transition | Collision energy/V | Identification transition | Collision energy/V |
| --- | --- | --- | --- | --- |
| 6.10 | 164/149 | 10 | 164/104 | 10 |

Table S2 Recovery, accuracy and precision of eugenol in feed samples (n = 6).

| spiking levels,  μg/kg | Recovery, (%) | | | | | | Average Recovery, % | RSD (intraday),  % | RSD (interday),  % |
| --- | --- | --- | --- | --- | --- | --- | --- | --- | --- |
| 0.4 | 107 | 95.4 | 99.4 | 102 | 98.3 | 105 | 101 | 4.29 | 4.19 |
|  | 114 | 110 | 116 | 99.5 | 108 | 115 | 110 | 5.59 |  |
|  | 117 | 113 | 87.2 | 98.6 | 106 | 110 | 105 | 10.3 |  |
| 2.0 | 92.6 | 95.1 | 94.1 | 93.4 | 92.8 | 98.1 | 94.4 | 2.17 | 8.41 |
|  | 109 | 111 | 112 | 115 | 107 | 113 | 111 | 2.57 |  |
|  | 93.0 | 89.7 | 103 | 95.4 | 101 | 92.1 | 95.7 | 5.48 |  |
| 4.0 | 89.0 | 86.6 | 88.5 | 87.6 | 84.8 | 89.9 | 87.7 | 2.09 | 7.75 |
|  | 95.0 | 93.6 | 87.4 | 92.3 | 94.2 | 88.5 | 91.8 | 3.43 |  |
|  | 99.9 | 95.8 | 105 | 102 | 110 | 96.8 | 102 | 5.24 |  |

Fig. S1 Chromatograms of eugenol in spiked fish feed sample (2 μg/kg).
